# The Brain–Gut Axis, an Important Player in Alzheimer and Parkinson Disease: A Narrative Review

**DOI:** 10.3390/jcm13144130

**Published:** 2024-07-15

**Authors:** Eugenio Caradonna, Raffaello Nemni, Angelo Bifone, Patrizia Gandolfo, Lucy Costantino, Luca Giordano, Elisabetta Mormone, Anna Macula, Mariarosa Cuomo, Rossana Difruscolo, Camilla Vanoli, Emilio Vanoli, Fulvio Ferrara

**Affiliations:** 1 Integrated Laboratory Medicine Services, Centro Diagnostico Italiano S.p.A., 20011 Milan, Italy; eugenio.caradonna@cdi.it (E.C.); fulvio.ferrara@cdi.it (F.F.); 2 Unit of Neurology, Centro Diagnostico Italiano S.p.A., Milan Fondazione Crespi Spano, 20011 Milan, Italy; raffaello.nemni@cdi.it; 3 Nuclear Medicine Unit, Imaging Department, Centro Diagnostico Italiano S.p.A., 20011 Milan, Italy; patrizia.gandolfo@cdi.it (P.G.); mariarosaria.cuomo@unimi.it (M.C.); 4 Department of Molecular Biotechnology and Health Sciences, University of Torino, 10124 Torino, Italy; angelo.bifone@unito.it; 5 Laboratory of Medical Genetics, Centro Diagnostico Italiano S.p.A., 20011 Milan, Italy; lucy.costantino@cdi.it (L.C.); luca.giordano@cdi.it (L.G.); 6 Institute for Stem-Cell Biology, Regenerative Medicine and Innovative Therapies (ISBReMIT), Fondazione IRCCS Casa Sollievo della Sofferenza, 71013 San Giovanni Rotondo, Italy; emormone@yahoo.com; 7 Centro Ricerche Bracco, Bracco Imaging S.p.A., Colleretto Giacosa, 10010 Turin, Italy; anna.macula@bracco.com; 8 Department of Physics, University of Torino, 10124 Torino, Italy; 9 Department of Oncology and Hemato-Oncology, University of Milan, 20122 Milan, Italy; 10 Faculty of Medicine, University of Bari, 70121 Bari, Italy; rossanadifruscolo@gmail.com; 11 Department of Clinical Psychology, Antioch University Los Angeles, Culver City, CA 90230, USA; 12 School of Nursing, Cardiovascular Diseases, University of Pavia, 27100 Pavia, Italy; emivano@gmail.com

**Keywords:** brain gut axis 1, TMAO, Alzheimer disease, Parkinson disease

## Abstract

Neurodegenerative diseases, such as Alzheimer’s disease (AD) and Parkinson’s disease (PD), are severe age-related disorders with complex and multifactorial causes. Recent research suggests a critical link between neurodegeneration and the gut microbiome, via the gut–brain communication pathway. This review examines the role of trimethylamine N-oxide (TMAO), a gut microbiota-derived metabolite, in the development of AD and PD, and investigates its interaction with microRNAs (miRNAs) along this bidirectional pathway. TMAO, which is produced from dietary metabolites like choline and carnitine, has been linked to increased neuroinflammation, protein misfolding, and cognitive decline. In AD, elevated TMAO levels are associated with amyloid-beta and tau pathologies, blood–brain barrier disruption, and neuronal death. TMAO can cross the blood–brain barrier and promote the aggregation of amyloid and tau proteins. Similarly, TMAO affects alpha-synuclein conformation and aggregation, a hallmark of PD. TMAO also activates pro-inflammatory pathways such as NF-kB signaling, exacerbating neuroinflammation further. Moreover, TMAO modulates the expression of various miRNAs that are involved in neurodegenerative processes. Thus, the gut microbiome–miRNA–brain axis represents a newly discovered mechanistic link between gut dysbiosis and neurodegeneration. MiRNAs regulate the key pathways involved in neuroinflammation, oxidative stress, and neuronal death, contributing to disease progression. As a direct consequence, specific miRNA signatures may serve as potential biomarkers for the early detection and monitoring of AD and PD progression. This review aims to elucidate the complex interrelationships between the gut microbiota, trimethylamine-N-oxide (TMAO), microRNAs (miRNAs), and the central nervous system, and the implications of these connections in neurodegenerative diseases. In this context, an overview of the current neuroradiology techniques available for studying neuroinflammation and of the animal models used to investigate these intricate pathologies will also be provided. In summary, a bulk of evidence supports the concept that modulating the gut–brain communication pathway through dietary changes, the manipulation of the microbiome, and/or miRNA-based therapies may offer novel approaches for implementing the treatment of debilitating neurological disorders.

## 1. Introduction

Alzheimer’s disease (AD) and Parkinson’s disease (PD) are the most common progressive neurodegenerative diseases, with diverse and largely unknown etiopathogeneses. AD is a major cause of dependence, disability and mortality. Current estimates suggest that 44 million people live with dementia worldwide at present. This is predicted to more than triple by 2050 as the population ages.

The incidence of PD in individuals over the age of 65 in North America ranges from 108 to 212 per 100 [1]. Within this same population, the estimated prevalence of PD in patients >45 years across North America is 572 per 100,000 [2]. This study projected that there will be 1,238,000 individuals with PD in the US by the year 2030.

Many environmental and lifestyle factors influencing the risk of AD and PD progression also affect the intestinal microbiota (e.g., diet and physical activity) [3,4], suggesting a plausible link between them.

Recent scientific evidence correlates alterations in the gut microbiota with neurodegenerative diseases, such as Alzheimer’s disease and Parkinson’s disease [5,6].

The intestinal microbiota, which comprises trillions of microorganisms that inhabit the gastrointestinal tract, plays a crucial role in regulating gut–nervous system interactions by producing neurotransmitters and other biologically active molecules that can enter systemic circulation.

The gut microbiota sends signals to the CNS and enteric nervous system through different pathways involving metabolites, hormones, the immune system, and afferent nerves, thus participating in the global metabolism of their host, including nutritional metabolism, immune function, and intestinal health [7,8].

The influence of the gut microbiota on neurodegeneration is mediated by its impact on neuroinflammation, protein aggregation, and neuronal signaling pathways. These effects are facilitated by mechanisms that operate along the gut–brain axis, involving the metabolic, immune, and neural pathways [9,10].

The apolipoprotein E (APOE) gene is the strongest and most prevalent genetic factor, impacting more than half of all AD cases. The ε4 allele of the APOE gene significantly increases AD risk, while the ε2 allele is protective relative to the common ε3 allele [11].

The biochemical and biophysical properties of apoE impact a cascade of events at the cellular and systems levels, ultimately impacting aging-related pathogenic conditions [12].

Of notice, APOE genotypes influence the L-carnitine system, necessary for fatty acid oxidation (FAO), and have a collective influence on the blood and brain. The APOE ε4 allele leads to an increased reliance on brain FAO compared to other genotypes, which could represent an earlier shift in the use of alternative energy substrates in APOE due to earlier impairments in glucose use [13,14]

In the research conducted by Huguenard et al., the plasma levels of L-carnitine, its metabolites γ-butyrobetaine and Trimethylamine-N- oxide (TMAO), and its esters (acylcarnitines) were examined in the APOE ε4- pre-clinical MCI/AD + MCI group. The findings showed that the ratios of these metabolites in the APOE ε4- group were similar to those of the control group. On the other hand, the profiles of the APOE ε4+ pre-clinical MCI/AD + MCI group were similar to those of the AD groups. This suggests that the peripheral profiles of these metabolites were altered earlier in the disease process among the APOE ε4 group compared to the non-ε4 group [15].

The gut plays a crucial role in the production of metabolites such as γ-butyrobetaine and TMAO. TMAO is a metabolite mainly produced in the liver through the oxidation of trimethylamine (TMA) by flavin monooxygenase 3 and, to a lesser extent, by flavin monooxygenase 1 [16]. TMA is metabolized by the gut microbiota from nutrients present in the Western diet, such as choline, lecithin, and carnitine.

The production of TMA and then TMAO (trimethylamine-N-oxide) is attributed to various species of intestinal bacteria, including Firmicutes, Actinobacteria, and Proteobacteria, as well as some non-commensal bacteria such as Burkholderia, Campylobacter, Aeromonas, Salmonella, Shigella, and Vibrio. Notably, the levels of TMAO in the blood increase with age, and high levels of TMAO have been correlated with a higher incidence and severity of cardiovascular diseases, diabetes, and certain types of cancers, such as colon cancer [17,18,19]. Furthermore, TMAO has been linked to Alzheimer’s disease [20,21,22,23].

Regarding PD, TMAO may prevent the aggregation of α-synuclein and the formation of insoluble fibers, which eventually induces PD [24]. α-Synuclein can aggregate into distinct fibril forms, termed polymorphs, which exhibit differences in their propensities to bind and penetrate cells, and in their toxicity and seeding properties. Braak’s staging of the PD brain pathology provides details about the staged distribution of deposition, with the progressive pattern of pathology correlating with the clinical aspects of the disease.

This concept has received extensive attention as it logically explains the clinical course of PD from the prodromal phase to dementia [25].

### 1.1. Alzheimer’s Disease

Alzheimer’s disease (AD) is the most common cause of dementia, accounting for approximately 80% of all dementia cases. It is characterized by the accumulation of tau protein tangles and amyloid β (β-amyloid peptide) plaques, progressive neuronal loss, and the deterioration of normal brain function. Typical AD presents as a progressive amnestic disorder with a specific episodic memory impairment profile characterized by low free recall that is not improved by cueing. The term Mild Cognitive Impairment (MCI) was introduced in the late 1980s by Reisberg et al. to characterize subjects at an intermediate stage on the Global Deterioration Scale (GDS). Peterson and colleagues refined the concept further by adding the memory complaint, recognizing that an awareness of decreasing mnemonic capabilities suggests that the subject is still at an early stage [14,15].

Subtyping MCI has been proposed to predict the development of dementia, according to the type and number of impaired cognitive domains (e.g., amnestic vs. non-amnestic MCI, and single vs. multiple-domain MCI) [15,16]. However, 30% of amnestic MCI cases (the most specific cases regarding AD phenotype) that progressed to dementia did not meet the neuropathological criteria for AD.

In 2016, the joint IWG-Alzheimer’s Association (IWG-AA) meeting furthered the integration of biomarkers into the definition of AD and decided to apply this definition independent of clinical status [26]. Indeed, the new definition of AD is now purely biological and is based on positivity for the biomarkers of both amyloidosis and tauopathy, independent of clinical status.

NIA-AA has recently formalized a new biological definition of AD. They proposed an A/T/N/C classification relying on CSF, PET, and MRI biomarkers (A = amyloid; T = tauopathy; N = neurodegeneration; C = cognitive change), where A and T positivity defines AD, whereas N and C are not specific to AD and define the severity stage of the disease. Hence, in line with the IWG-AA 2016 document, the focus of these criteria is no longer on the symptoms, but on the biological in vivo definition of the disease. In line with the conceptual evolution, tauopathy might not only be the downstream consequence of amyloid pathology, but also a parallel and independent pathological process [27,28]. The hierarchy between amyloid and tau biomarkers has been softened, and concomitant tauopathy and amyloidosis now represent AD. The amyloid biomarkers validated by these criteria are CSF Aβ42 or the Aβ42/Aβ40 ratio and amyloid-PET.

Biomarker changes that occur in the 20-year period between normal cognition and the diagnosis of sporadic Alzheimer’s disease have been investigated in a study involving Chinese participants. CSF and the imaging biomarkers in the Alzheimer’s disease group diverged from those in the cognitively normal group. As cognitive impairment progressed, the changes in the CSF biomarker levels in the Alzheimer’s disease group initially accelerated and then slowed [29].

When no biomarker was available, the 2018 NIA-AA criteria introduced the concept of Alzheimer’s clinical syndrome, which applies to both mildly impaired and demented individuals. This refers to the definitions of possible and probable AD according to previous NINCDS-ADRDA12 and NIA-AA46 criteria. Nonetheless, little is known about the precise phenotype, except for a vague “multi-(or single-) domain amnestic syndrome” or a “classic syndromic” variant.

Another distinction that must be made is between early-onset AD (EOAD) and late-onset AD (LOAD), defined by the individuals’ age at the first manifestation of symptoms (< or >65 years). LOAD represents the majority of AD cases (>95%) 72.73. EOAD remains the most common cause of early-onset neurodegenerative dementia. In contrast to LOAD, which is a complex disorder with a heterogeneous etiology and a 70–80% heritability (according to some models), EOAD is almost entirely genetically determined, with a heritability ranging between 92% and 100% 74.75. Between 35–60% of EOAD patients have at least one affected first-degree relative, and in 10% to 15% of those with familial EOAD, the mode of inheritance is autosomal dominant [30]

The pathophysiology of AD is considered a molecular–metabolic dysfunction within the brain, in which the formation of beta amyloid plaques (Ab) and neurofibrillary tangles caused by the aggregation of beta-amyloid peptides and tau proteins, respectively, appear to be the key features involved. However, treatment with solanezumab (an Ab-targeted antibody) in patients with AD has failed to improve cognitive decline [31].

### 1.2. Parkinson’s Disease

PD is a condition that is marked by the loss of neurons in the substantia nigra pars, which is associated with the accumulation of ubiquitinated α-synuclein and other proteins in cytoplasmic inclusions. There is evidence that suggests a clear link between TMAO and PD, as TMAO is known to cause α-synuclein peptide to convert into compact and folded conformations. The conformational change in monomeric α-synuclein, which results in fibrillary aggregation, is a crucial pathological mechanism of PD. TMAO can regulate the conformational properties of the α-synuclein peptide structure, as presented in this concept.

The process of aggregation generates harmful intermediate products, such as protofibrillar and oligomeric forms, which can impair the functioning of lysosomes, proteasomes, or mitochondria; damage the cytoskeleton and biological membranes; and alter synaptic function, resulting in the loss of neurons, particularly in the substantia nigra pars compacta (SNpc) region [32]. In the early stages of PD, the Lewy body pathology affects specific regions, usually limited to the olfactory bulb, and may also impact the intermediate reticular zone or dorsal nucleus of the vagus nerve. As the disease advances, the pathology becomes evident in the midbrain, affecting the SNpc. Furthermore, there is growing evidence that α-syn fibrils can be transmitted in a prion-like manner between cells, contributing to the progression of the disease [33]. The SNpc contains dopaminergic neurons that degenerate as the disease progresses, leading to the manifestation of both motor and non-motor symptoms.

## 2. Animal Model

Murine models of Alzheimer’s and Parkinson’s disease are pivotal in the study of the molecular mechanisms underlying these pathologies and for the validation of novel therapeutics. These models mimic the complex pathophysiology of these neurodegenerative diseases in a controlled environment, facilitating a detailed investigation of the disease progression and underlying molecular processes. By replicating key aspects such as amyloid plaque formation in Alzheimer’s and dopaminergic neuron degeneration in Parkinson’s, murine models provide critical insights into disease mechanisms. Moreover, they serve as essential platforms for testing the efficacy and safety of potential therapeutic interventions, enabling the translation of basic research findings into clinical applications.

### 2.1. Murine Models of Alzheimer’s Disease

In the following, we summarize murine models that may be useful for testing mechanisms that involve enzymes promoting the aggregation of beta-amyloid in Alzheimer’s disease (AD). These models are engineered to express how human amyloid precursor protein (APP) leads to amyloid plaque formation, cognitive deficits, and other AD-like pathologies. Recent efforts have been directed toward creating models that encompass a broader range of AD pathologies, thereby improving their relevance for human AD.

Traditional transgenic models of AD include the APPswe (Swedish Mutation) Mice. These mice carry a mutation in the APP gene that leads to the increased production of amyloid-beta (Aβ) peptides. The Swedish mutation (KM670/671NL) significantly enhances the β-secretase cleavage of APP, leading to an accumulation of Aβ plaques. The seminal paper by Hsiao et al. [34] describes the development of the Tg2576 transgenic mouse model, which expresses a mutant form of human amyloid precursor protein (APP) with the Swedish mutation. These mice develop amyloid plaques and cognitive deficits, and represent a foundational model for AD research.

One of the key developments in this area is the creation of second-generation mouse models. These models often incorporate multiple genetic mutations known to be associated with familial forms of Alzheimer’s disease [35]. APP/PS1 mice combine mutations in both the APP gene and the presenilin 1 (PS1) gene [36]. The PS1 mutation affects γ-secretase, an enzyme complex involved in APP processing. These mice show earlier and more robust Aβ plaque formation and cognitive impairments compared to single-transgenic models. 5xFAD mice harbor five familial AD mutations (three in APP and two in PS1), leading to an early and aggressive amyloid pathology. They could prove highly useful for studying the role of plaque-stabilizing enzymes in both the early and late stages of amyloid deposition [37].

Inducible models of APP mutant mice have been developed using the Tet-Off strategy [38]. These models use a tetracycline-responsive promoter element to control the expression of mutant APP. When bred to transgenic mice expressing reverse tertracycline-controlled transactivator protein (rtTA) or tetracycline-controlled transactivator protein (tTA), the expression of the APP transgene can be turned off with the tetracycline analog, doxycycline, in bigenic animals. This allows researchers to temporally control the onset of amyloid pathology by administering or withdrawing doxycycline, making it possible to study the effects of therapeutic interventions at specific disease stages.

Overexpressed APP results in the overproduction of Aß, but also in a variety of APP fragments that could contribute confounding pathophysiological effects to the model [39]. To overcome these drawbacks, mouse models of sporadic AD have been developed using a knock-in strategy to introduce the Swedish mutation, which increases all Aß species, into the APP gene, together with either the Beyreuther/Iberian mutation or the Beyreuther/Iberian plus the Arctic mutation [40,41]. These second-generation models express normal APP levels but develop robust Aß pathology, neuroinflammation, and memory impairment, which are hallmark features of AD. The latter line is particularly noteworthy as it develops Aβ pathology approximately three times faster, making it a valuable tool for studying downstream events like neuroinflammation, oxidative stress, tau propagation, and spatial memory impairment.

Murine models of Alzheimer’s disease (AD) based on presenilin (PSEN) genes, particularly PSEN1 [42,43] and PSEN2 [44,45], have also been extensively investigated for studying the pathogenesis and progression of familial Alzheimer’s disease (FAD). For instance, models such as the APP/PS1 have been extensively used to observe the age-dependent progression of these pathologies and their effects on cognitive functions [46,47]. These studies have been pivotal in demonstrating how PSEN mutations not only lead to typical AD symptoms, but also how these symptoms vary significantly with age and between genders, reflecting the complex interaction of genetic and environmental factors in disease manifestation. In addition to amyloid and tau pathologies, murine models with PSEN mutations also exhibit other significant biochemical changes. For example, alterations in glutamate transport and synaptic dysfunction due to impaired presenilin function have been shown to contribute to neurodegeneration. Studies such as those by Salcedo et al. and Jęśko et al. [48,49] have explored how presenilin mutations affect neuronal communication by disrupting calcium signaling and synaptic protein expression, leading to abnormal synaptic activity and neuronal death. Moreover, these murine models have also facilitated the exploration of novel therapeutic approaches. For example, the potential neuroprotective effects of compounds like Fingolimod (FTY720) have been tested in these models, showing the reversal of some of the pathological changes induced by APP and PSEN mutations, particularly in older mice, indicating age-dependent therapeutic effects [49].

Furthermore, recent research has highlighted the role of presenilin in DNA repair mechanisms within the brain. For instance, Authiat et al. [50] discovered that the PS1 P117L Alzheimer model shows lower levels of DNA double-strand breaks, suggesting a neuroprotective role for the BRCA1/BARD1 pathway in neurons with presenilin mutations. This insight opens up new avenues for understanding the cellular mechanisms that might protect against or exacerbate Alzheimer’s disease, potentially leading to novel therapeutic targets.

Current research (summarized, e.g., in [51]) focuses on the development of models that explore synergistic factors to develop models that exhibit a more cored plaque pathology. A mutant Psen1 knock-in mouse carrying a pathogenic mutation (P117L) has been used to generate a new model that exhibits the early deposition of wild-type human Aβ, demonstrating the synergistic interaction between APP and PSIN1 genes [52]. These novel mutant mice (sometimes referred to as 3rd generation models) provide powerful tools for examining the pathologic mechanisms upstream of Aβ deposition, and can be useful for the preclinical screening of disease-modifying therapy candidates promoting Aβ degradation or disaggregation,

In summary, the recent advances in murine models for Alzheimer’s disease research have greatly enhanced our ability to study the disease in a manner that is more representative of the human condition. These new models, with their specific genetic modifications and resultant pathologies, offer a more comprehensive understanding of the disease and a better platform for testing potential treatments. Importantly, the models summarized above provide a means to study the effects of TMAO on a variety of aspects of Alzheimer’s, including the stage (early vs. late), amyloid deposition, neuroinflammation, and synaptic dysfunction.

### 2.2. Murine Models of Parkinson’s Disease

The complexity of PD pathogenesis necessitates the use of animal models to understand the underlying mechanisms and to develop effective treatments. Murine models, particularly those using mice, have been invaluable in this research due to their genetic manipulability, short reproductive cycles, and physiological similarities to humans. This essay briefly delves into the various murine models of PD employing different experimental approaches, highlighting their contributions and limitations.

## 3. Genetic Models

### 3.1. α-Synuclein Overexpression Models

The hallmark of PD pathology is the presence of Lewy bodies, primarily composed of α-synuclein. Transgenic mice overexpressing human α-synuclein (SNCA) exhibit many PD-like features, including progressive motor deficits and dopaminergic neuron loss. The A53T mutation, associated with familial PD, has been particularly useful. Mice carrying this mutation develop motor impairments and neurodegeneration, mimicking human PD progression (Dawson et al., 2010) [53]. However, these models often do not fully recapitulate the selective loss of nigral dopaminergic neurons seen in human PD.

### 3.2. LRRK2 Models

Mutations in the LRRK2 gene are the most common genetic cause of familial PD. Mice carrying the G2019S mutation in LRRK2 develop age-dependent motor deficits and dopaminergic neuron loss (Cookson, 2015) [54]. These models are particularly valuable for studying the molecular pathways involved in PD and for testing LRRK2 kinase inhibitors as potential therapies.

### 3.3. DJ-1, PINK1, and Parkin Models

Recessive mutations in the genes encoding DJ-1, PINK1, and Parkin are linked to early-onset PD. Mice deficient in these genes exhibit mitochondrial dysfunction and increased oxidative stress, reflecting some aspects of PD pathogenesis [55]. However, they do not typically show significant dopaminergic neuron loss or Lewy body formation, limiting their use in modeling the full spectrum of PD pathology.

### 3.4. Toxin-Induced Models

#### 3.4.1. MPTP Model

The neurotoxin MPTP is one of the agents most widely used to induce PD-like symptoms in mice. MPTP crosses the blood–brain barrier and is metabolized to MPP+, which selectively targets dopaminergic neurons. MPTP-treated mice exhibit rapid and severe nigrostriatal degeneration, mimicking the dopaminergic neuron loss seen in PD (Jackson-Lewis and Przedborski, 2007) [56]. While this model is excellent for studying neurodegeneration mechanisms, its acute nature does not reflect the progressive nature of human PD.

#### 3.4.2. 6-OHDA Model

6-Hydroxydopamine (6-OHDA) is another neurotoxin used to induce dopaminergic neuron degeneration. The injection of 6-OHDA into the substantia nigra or striatum of mice leads to selective dopaminergic neuron loss and motor deficits. This model is useful for studying the effects of dopaminergic neuron loss and for evaluating neuroprotective therapies [57]. However, similar to the MPTP model, it does not fully replicate the chronic and progressive aspects of PD.

### 3.5. Inflammatory Models

#### 3.5.1. LPS Model

Lipopolysaccharide (LPS), a component of the outer membrane of Gram-negative bacteria, induces strong inflammatory responses. The intraparenchymal injection of LPS into the substantia nigra of mice triggers microglial activation and selective dopaminergic neuron loss. This model is valuable for studying the role of neuroinflammation in PD pathogenesis.

#### 3.5.2. Limitations and Future Directions

While murine models have significantly advanced our understanding of PD, they each have inherent limitations. Genetic models often do not exhibit the full spectrum of PD pathology, and toxin-induced models fail to capture the progressive nature of the disease. Additionally, differences in brain structure and function between mice and humans can limit the translatability of findings.

Future research should focus on developing more sophisticated models that combine genetic predispositions with environmental factors to better mimic the multifactorial nature of PD. The advent of CRISPR/Cas9 technology and the generation of humanized mouse models hold promise for creating more accurate models of PD. Moreover, integrating multi-omics approaches with murine models can provide deeper insights into the complex interactions underlying PD pathogenesis.

In summary, murine models of Parkinson’s disease have been instrumental in unraveling the molecular and cellular mechanisms of the disease and in testing potential therapeutic strategies. While each model has its strengths and limitations, together they provide a comprehensive toolkit for PD research. The continued refinement and development of these models will be crucial for translating basic research findings into clinical applications, ultimately improving outcomes for patients with PD.

## 4. Neuroinflammation and Nuclear Imaging

Neuroinflammation is a complex biological response to various forms of neural injury or disease, characterized by the activation of the brain’s innate immune system, particularly microglia, the resident immune cells of the central nervous system (CNS). These cells are responsible for maintaining CNS homeostasis, defending against pathogens, and repairing tissue damage. Their activation appears to have a protective aim as they act to clear pathogens and debris. However, excessive or chronic activation could lead to neural damage and contribute to the progression of different neuronal disorders instead of aiding in their resolution [58].

The glial activation process involves the release of different molecules, including pro-inflammatory cytokines and chemokines, and the overexpression of proteins, such as the translocator protein (TSPO). The mechanisms of glial activation are influenced not only by the CNS but also by external factors, such as the gut microbiota, which plays a fundamental role in microglial maturation, identity, and function. The gut–brain axis is regulated by a series of immune, enteric, and neural pathways that change with age. Abnormal alterations can lead to systemic inflammatory processes, including an excessive increase in the permeability of the gut epithelial barrier. This phenomenon causes the leakage of bioactive molecules, such as short-chain fatty acids (SCFAs), kynurenines, melatonin, histamine, bile acids, and neurotransmitters, into the bloodstream.

Since liver cannot remove all circulating biomolecules, these can more easily cross the blood–brain barrier (BBB), facilitated by the fact that it becomes more permeable with age, and cause neuroinflammation and macrophage dysfunction [59,60]. The dysregulation processes in both the CNS and gut are intrinsically correlated, as evidenced by numerous studies on microbiota alterations in neurodegenerative injuries, such as Alzheimer’s disease (AD) and Parkinson’s disease (PD).

Glial activation has been distinguished into proinflammatory or anti-inflammatory types, probably depending on the stage of the disease. For example, in Alzheimer’s disease, the relationship between microglial activation and amyloid-beta (Aβ) plaques is not clear. On one hand, evidence suggests that neuroinflammation is an early event in AD because the cytokines produced by microglia can upregulate the production of beta-secretase, an enzyme responsible for generating pathogenic Aβ. On the other hand, an in vivo study demonstrated the role of microglia in limiting the diffusion of amyloid plaques [61,62]. Neuroinflammation also plays a fundamental role in Parkinson’s disease, constituting a potential marker for its diagnosis and treatment. However, its dual role, both promoting and inhibiting PD onset, remains to be clarified. The oligomerization and aggregation of the α-Synuclein (α-Syn) protein seem to be the first step in the cascade of PD pathogenesis. These aggregates accelerate damage to dopaminergic (DA) neurons by activating inflammatory processes, concurrently inducing microglia to activate NLRP3 inflammasomes and obstructing microglial autophagy and phagocytosis. Conversely, the autophagic capacity of microglia can inhibit the α-Syn aggregation process by phagocytosing both α-Syn fibrils and activated NLRP3 inflammasomes [63]. All these conflicting pieces of evidence and the complex mechanism of neuroinflammation, which can act as both a promoter and inhibitor, make it evident that further investigation into the role and dynamics of microglial activation in neurodegenerative pathologies is warranted.

Molecular imaging could play a crucial role in characterizing the early stages of the disease to identify an appropriate time window for targeted therapy.

Positron Emission Tomography (PET) imaging provides insights into the real-time biological processes at the molecular level based on a specific ligand bearing a positron-emitting radionuclide (“PET tracer”), which confers this technology high sensitivity and excellent tissue penetration.

This technique is the preferred imaging method for quantifying neuroinflammation, thanks to its ability to detect selected proteins at low concentrations. However, the measurement of inflammation processes in the brain is not yet employed in routine clinical practice due to several limitations, such as the complexity of their quantification and the lack of data on large patient samples, thus limiting their current use in research.

Among the different molecules that are involved in neuroinflammation, the mitochondrial 18 kDa translocator protein (TSPO) is the most widely investigated target. Many studies have indeed demonstrated TSPO upregulation in patients affected by AD and in animal models of AD [64,65], but there is further evidence of a TSPO increase also in patients affected by Parkinson’s Disease [66].

This protein is mainly expressed in the outer mitochondrial membrane of steroid-synthesizing cells in the central nervous system and in the periphery [67]. Its expression is strongly upregulated in activated microglial cells by inflammatory stimuli. Although the concentration of TSPO is prevalent in brain microglia, it is also expressed by astrocytes and vascular endothelium, which could lead to the misinterpretation of PET imaging due to the relative contribution of TSPO radioligand binding by microglia versus other cells, depending on the disease studied.

Many radioligands have been developed to bind TSPO molecules. The first generation of TSPO tracers could be represented by [11C]PK-11195, which has been employed in many in vivo and clinical studies. However, these tracers suffer from a low signal-to-noise ratio due to their poor physical characteristics, such as low blood–brain barrier permeability and high nonspecific plasma binding [68]. Therefore, there was a need for the development of second-generation radioligands, such as [11C]-PBR28, [18F]-FEPPA, [18F]-DPA714, and [11C]-DPA713 [69]. These newer radioligands targeting TSPO have a better signal-to-noise ratio than (R)- [11C]-PK11195, but they show wide variability among subjects due to a single nucleotide polymorphism (SNP) (rs6971) in the gene encoding TSPO [8, XX1]. This rs6971 polymorphism causes differences in the binding affinity of radioligands to the translocator protein (TSPO). For this reason, patients can be categorized according to their genotype into high-affinity binders (HABs), mixed-affinity binders (MABs), and low-affinity binders (LABs).

In recent years, third-generation radioligands, such as [11C]ER-176 and [18F]-GE-180, have been developed to reduce inter-patient binding variability, decreasing the binding sensitivity to the TSPO polymorphism. There are even examples of radioligands, such as [11C]GE-180 and [11C]GE-387, which are completely insensitive to the polymorphism in in vitro tests, allowing for more consistent and reliable PET imaging across different genotypes. However, further in vivo examination is still needed to ensure their neutrality [61].

Even if the binding variability seems to be resolved, TSPO targets are affected by other limitations in terms of quantification. Indeed, TSPO is not exclusively expressed by microglial cells but also by other cell types within the central nervous system, such as activated astrocytes, peripheral macrophages, and endothelial cells. As a consequence, eventual TSPO upregulation by PET imaging could be attributable not only to neuroinflammation processes due to microglial cell activation, but also to other physiological problems, leading to the difficult interpretation of PET imaging results. Moreover, there is not any reference region that could act as a “baseline” uptake of TSPO.

Several other molecular targets implicated in neuroinflammation have been identified as potential alternatives to TSPO, to obtain biomarkers that are more sensitive and specific [61]. Monoamine Oxidase B (MAO-B), an enzyme expressed by astrocytic cells, could be a possible target for neuroinflammation processes. Currently, only 11C- deuterium-l-deprenyl (11C-DED) has been used to characterize astrocytic activation in the CNS, and there is evidence that 11C-DED is present in the prodromal stages of AD [70]. Another promising target is cyclooxygenase, an enzyme involved in the production of prostaglandin H2. The two isoforms COX-1 and COX-2 are expressed in microglia and neurons in the CNS, and are involved in neuroinflammation in neurodegenerative diseases. Several tracers have been developed for these molecules, including [18F]TMI [71], [11C]KTP-Me [72,73,74], [11C]PS13, and [11C]MC1 [75,76], which are under in vivo and clinical studies. Limited evidence exists of increased brain uptake in AD patients compared to healthy controls using the [11C]KTP-Me tracer.

Purinergic receptors (P2X7 and P2Y12) are also excellent candidates for neuroinflammation targets, because of their upregulated expression specific to microglia cells. Some receptor tracers, such as [11C]P2Y12R-ant and [11C]5, have already been evaluated in in vivo studies, particularly using an autoimmune encephalomyelitis model of multiple sclerosis [75] and a stroke model [77], but they have not yet been evaluated in MCI and AD models.

Neuroinflammation is instigated by the misfiring of immune cells in the central nervous system (CNS), involving microglia and astrocytes as key cell types, and nuclear imaging implementation provides a critical contribution to neurodegenerative disorder detection and management

### 4.1. Diagnostic and Therapeutic Implementation: The Role of TMAO

#### 4.1.1. TMAO and Alzheimer’s Disease [1]

Trimethylamine-N-oxide (TMAO) has been linked to Alzheimer’s disease (AD) in several studies.

TMAO is associated with Alzheimer’s disease through various pathophysiological pathways, including the aggregation of amyloid-beta peptide and tau protein, which are key in the development of Alzheimer’s disease pathology. TMAO plays a crucial role in stabilizing the compact monomer geometries of intrinsically disordered proteins (IDPs), such as tau protein. By promoting folding and reducing the formation of certain secondary structures, TMAO achieves stabilization without significantly affecting the formation of helical monomers. This mechanism is achieved through a subtle process in which TMAO acts as a molecular crowder and is depleted from the protein surface as the protein adopts its native state. TMAO interacts more with water molecules than with neighboring TMAO molecules, forming a complex with 2–3 water molecules, which leads to a wider hydration shell around TMAO than around urea. This interaction pattern affects the hydration and stabilization of proteins, with TMAO favoring the formation of compact oligomers, including helical oligomers, through a mechanism that involves the redistribution of water around the perimeter of the peptide [78].

**Figure 1:**
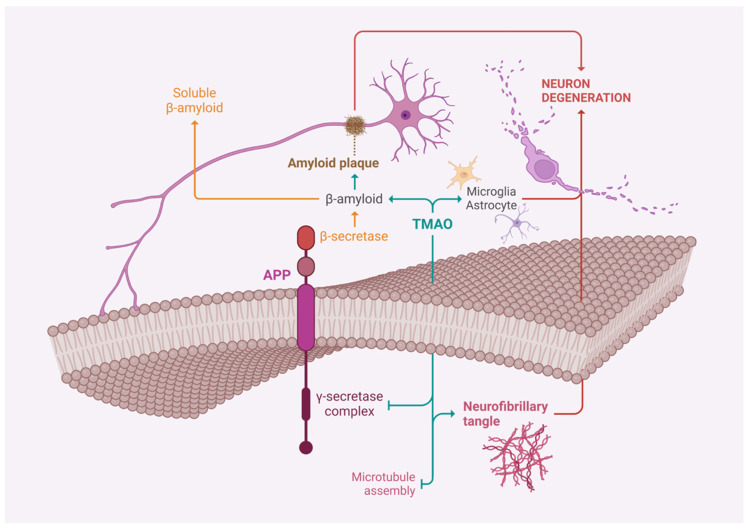
TMAO action to induce Alzheimer’s Disease.

Additionally, TMAO can activate astrocytes and inflammatory responses, further supporting its role in cognitive decline and Alzheimer’s disease [79] (Figure 1).

Xu et al. found that, among 56 microbiota-derived metabolite markers, TMAO demonstrated the strongest predictive correlation with memory and cognitive changes in AD. This correlation was established through an integrated analysis of genetic, epigenetic, pathological, and biochemical data [80].

The presence of TMAO in cerebrospinal fluid confirms its ability to cross the blood–brain barrier. Approximately 26% of TMAO is passively diffused through the blood–brain barrier (BBB) after 12 h of perfusion [81].

The TMAO physiological values in cerebrospinal fluid range between 0.11 and 6.45 mol/L, and it appears to exert a stabilizing and protective action on the blood–brain barrier within these parameters [82]. However, when TMAO concentrations exceed these levels, they accelerate the conversion of amyloid β (Aβ) fibrils and stabilize their aggregation into plaques [83]. In addition, TMAO induces the formation and consolidation of multiple tau conformers [84]. Notably, the levels of TMAO in the cerebrospinal fluid are strongly correlated with plasma levels [85].

TMAO also affects the blood–brain barrier by inhibiting proteins that regulate junctions, specifically claudin-5 and zonula occludens-1 [81]. This was demonstrated in studies showing that high TMAO levels are associated with microangiopathy, leading to decreased cerebral perfusion and brain degeneration [81,86,87].

Another critical element in AD is clusterin that, in its predominant form, is a secreted heterodimeric glycoprotein of 75–80 kDa [88] and is expressed in almost all mammalian tissues.

Within the brain, clusterin is the second most abundantly expressed apolipoprotein putatively involved in Alzheimer’s disease, and it is significantly correlated with progression [89,90].

The key factor is that TMAO increases clusterin production, thus further activating neuroinflammation [91].

Brunt et al. established that TMAO substantially influences neuroinflammation and cognitive decline in an aging population [85]. The study indicated that among middle-aged and elderly participants (average age, 65 years), higher plasma TMAO levels were associated with a poorer performance in cognitive tests, specifically those assessing memory and fluid cognition, using the NIH Toolbox.

In the experimental part of the study carried out in mice, the TMAO levels corresponded linearly with astrocyte activation and the neuroinflammatory state [85].

During the course of this research, it was found that a decrease in the levels of TMAO in animals resulted in an improvement in cognitive function and a reduction in the accumulation of amyloid-beta plaques, which is a typical expression of AD.

In a comprehensive study conducted by Vogt et al., the relationship between TMAO and AD was investigated by analyzing the concentrations of TMAO in CSF obtained from a representative sample of participants, comprising individuals with clinical Alzheimer’s syndrome (*n* = 40), individuals with MCI (*n* = 35), and cognitively unaffected individuals (*n* = 335). The sample size of this study was appreciable and comprised a diverse demographic spectrum. The statistical analysis indicated that the concentration of TMAO in the CSF was higher in individuals with MCI and AD dementia than in those who were cognitively unimpaired. Additionally, elevated levels of TMAO in CSF are correlated with the presence of biomarkers associated with AD pathology, such as phosphorylated tau and phosphorylated tau/A β42, as well as biomarkers of neuronal degeneration, such as total tau and neurofilament light chain protein [92].

The mammalian (or mechanistic) target of rapamycin (mTOR) is a critical cell signaling pathway that plays a vital role in various physiological functions, such as cell growth, proliferation, metabolism, protein synthesis, and autophagy [93]. Dysregulation of the mTOR pathway has been associated with the pathophysiology of neurological diseases, particularly AD [94,95].

In mice treated with TMAO, mTOR is profoundly altered [96]. Mitochondria play a pivotal and central role in ensuring optimal neuronal and synaptic functions, both of which are impaired in AD. Mitochondrial malfunction is one of the principal causes of AD [97] and, once more, TMAO participates in mitochondria dysfunction, thus further favoring oxidative stress [91].

#### 4.1.2. TMAO and Parkinson Disease

There is growing evidence that the dysfunction of the microbiota–gut–brain axis is an important trigger underlying the pathogenesis of Parkinson’s disease (PD) [98,99,100]. According to recent studies, an increase in TMAO levels has been observed in patients with PD, and this increase has been found to be associated with the severity of the disease and the progression of motor symptoms [22].

In a previous study, plasma TMAO levels were lower in patients with drug-naïve early-stage PD. Additionally, baseline plasma TMAO levels were associated with faster increases in the longitudinal requirement of dopaminergic medication and tended to increase the risk of PD–dementia conversion, suggesting the potential prognostic implications of TMAO in the early stage [101].

The application of methylamine TMAO in investigations pertaining to α-synuclein, a protein linked to Parkinson’s disease, has shown its capacity to facilitate the conversion of misfolded proteins into their native state. In circumstances where typical levels of TMAO are present, α-synuclein exhibits a more physiological helical conformational arrangement [102].

Low levels of TMAO may lead to the formation of partially folded α-synuclein in a non-physiological state. This finding and the natural role of TMAO suggest the hormetic effect of this molecule [103]. The existing evidence from PD models has indicated high TMAO levels have potential detrimental effects [104,105].

The plasma levels of bacteria-derived metabolites, including TMAO, short-chain fatty acids, the branched-chain fatty acid isovalerate, succinate, and lactate, were evaluated in PD subjects (treatment-naïve and treated), which were compared to (1) population controls, (2) spousal/household controls (similar lifestyle to PD subjects), and (3) subjects with multiple system atrophy. The analyses revealed an increase in the TMAO pathway in subjects with PD, which was independent of medication status, disease characteristics, and lifestyle. Lactic acid was decreased in treated PD subjects, succinic acid was positively correlated with disease severity, and the ratio of pro-inflammatory TMAO to the putative anti-inflammatory metabolite butyric acid was significantly higher in PD subjects than in controls, indicating a pro-inflammatory shift in the metabolite profile of PD subjects [106].

In summary, TMAO engages in the processes of AD and PD and results in a systemic inflammatory state, which also contributes to the development of cardiovascular disease, diabetes, and cancer [107,108,109]. The relationship between TMAO levels and frailty/mortality in elderly individuals is well established [110,111].

The preclinical identification and management of disease progression through the integration of biomarkers, such as TMAO, into diagnostic pathways can play a significant role in complementing the initial assessment and patient evaluation process. This approach can help refine the complex therapeutic interventions for patients with chronic conditions.

### 4.2. The Interplay between MicroRNAs, the Gut Microbiota, and the Brain-Gut Axis in Neurodegenerative Diseases

Recent studies have shed light on the complex interaction between microRNAs (miRNAs), the human gut microbiota, and a network known as the brain–gut axis in the pathogenesis of these debilitating conditions [112,113]. Moreover, emerging evidence suggests that gut microbiota-derived metabolites, such as short-chain fatty acids (SCFA) and lipopolysaccharide (LPS), modulate miRNA expression and function in the CNS.

The gut microbiota consists of trillions of microorganisms that are essential for digestion, metabolism, immune function, and protection against pathogens. The composition of the gut microbiota is influenced by diet, lifestyle, and genetics. The brain–gut axis bidirectionally links the central nervous system (CNS) and the gastrointestinal (GI) tract, involving neural, hormonal, and immunological signaling pathways. Key components include the vagus nerve, the enteric nervous system, the hypothalamic–pituitary–adrenal (HPA) axis, and the gut microbiota. The miRNAs can be secreted into the gut lumen and influence the composition and function of the gut microbiota. For instance, miRNAs from host cells can enter bacterial cells and affect their gene expression, thereby modulating bacterial growth and activity. Gut bacteria can influence the host’s miRNA expression. Certain bacterial metabolites like short-chain fatty acids (SCFAs) can modulate the expression of miRNAs in the host. This can have downstream effects on gene regulation in the gut and other tissues.

Indeed, SCFAs can modulate miRNA expression in the gut epithelium. For example, butyrate can influence the expression of miRNAs involved in gut motility and pain perception, potentially affecting Irritable Bowel Syndrome (IBS) symptoms. In addition, butyrate has been shown to upregulate miR-223, which has anti-inflammatory effects by targeting and downregulating the NLRP3 inflammasome, a component involved in inflammatory responses. In fact, dysregulated miRNA expression has been linked to IBS; miRNAs can affect the expression of serotonin receptors and other proteins involved in gut motility and sensitivity, playing a role in the pathophysiology of IBS [114,115,116].

Gut bacteria produce metabolites (e.g., SCFAs, tryptophan metabolites) and neuroactive compounds (e.g., serotonin, gamma-aminobutyric acid GABA) that can influence the CNS. These compounds can cross the blood–brain barrier (BBB) or signal through the vagus nerve. The gut microbiota can modulate the immune system, influencing neuroinflammation, which is a key feature of many neurodegenerative diseases. Dysbiosis, or an imbalance in the gut microbiota, can lead to systemic inflammation that affects the brain. The vagus nerve serves as a major communication pathway between the gut and the brain. Signals from the gut microbiota can influence brain function and behavior through vagal afferents. miRNAs are involved in the regulation of neurotransmitter systems, synaptic plasticity, and neuronal survival. The dysregulation of specific miRNAs can lead to neural dysfunction, contributing to the pathogenesis of neurodegenerative diseases. miRNAs can be packaged into extracellular vesicles (e.g., exosomes) and circulate in the bloodstream, mediating communication between the gut and the brain. These circulating miRNAs can cross the BBB and affect neuronal gene expression.

The disruption of the brain–gut axis, through alterations in the gut microbiota composition or aberrant miRNA expression, can contribute to the pathogenesis of neurodegenerative diseases [117,118,119,120]. In addition, dysbiosis of the gut microbiota has been associated with alterations in miRNA expression and function, contributing to the pathogenesis of neurodegenerative diseases.

miRNAs interact with chaperones like Hsp70 (Heat shock protein 70) in ways that are crucial for cellular function, particularly in the context of neurodegenerative diseases. Hsp70 is a highly conserved molecular chaperone involved in protein folding, repair, and degradation. It plays a pivotal role in maintaining protein homeostasis (proteostasis) by preventing protein aggregation, a common feature in neurodegenerative diseases. Hsp70 can influence the expression of miRNAs. For instance, during cellular stress, Hsp70 can modulate the levels of specific miRNAs that regulate stress response genes [116,121].

Conversely, the modulation of the gut microbiota through dietary interventions, probiotics, or fecal microbiota transplantation has been shown to attenuate neuroinflammation and improve cognitive functions involving miRNAs action [122,123,124].

In fact, miRNAs can also regulate the expression of Hsp70. Certain miRNAs bind to the mRNA of Hsp70, controlling its translation and thus its levels in the cell.

Moreover, several miRNAs can regulate gene expression at the epigenetic level by targeting components such as histone-modifying enzymes or DNA methyltransferases. This can lead to changes in the chromatin structure and gene accessibility, ultimately affecting the expression of genes involved in various pathological conditions including neurodegeneration. Certain miRNAs use these epigenetic modifications to exert tight control over the inflammatory response in nervous tissue [125,126,127]. The dysregulation of microRNAs (miRNAs) is believed to contribute significantly to the development of various neurodegenerative conditions including AD, PD, multiple sclerosis (MS), and amyotrophic lateral sclerosis (ALS). These diseases are associated with the continuous deterioration of neuronal function and a loss of structure, culminating in cognitive impairment, motor deficits, and other disabling symptoms. Mounting evidence suggests that the dysregulation of miRNAs contributes to the pathogenesis of these disorders by modulating the gene expression networks involved in inflammation, oxidative stress, and neuroprotection [128,129].

MicroRNAs (miRNAs) are known to exert their molecular effects through various mechanisms. One such mechanism involves binding to the 3-untranslated region (3-UTR) of target messenger RNAs (mRNAs), leading to their degradation. As a result, the translation of mRNA into protein is prevented, which ultimately reduces the expression of proinflammatory genes associated with neuroinflammation. In addition, certain miRNAs can impede the translation of target mRNAs into proteins by interfering with ribosome assembly or stability, thereby suppressing the expression of inflammatory mediators. Furthermore, miRNAs are involved in feedback loops that regulate the expression of transcription factors and signaling molecules involved in neuroinflammation. By targeting these regulators, miRNAs can modulate the intensity and duration of the inflammatory response [130,131,132].

The modulation of various signaling pathways involved in neuroinflammation by microRNAs (miRNAs) is an essential aspect of their functions. Specifically, miRNAs can directly target the components, such as receptors, signaling molecules, and transcription factors, involved in these pathways, including the nuclear factor NF-kB pathway. The NF-kB pathway plays a crucial role in the expression of pro-inflammatory genes in response to various stimuli, including cytokines (e.g., TNF-α and IL-1β), pathogen-associated molecular patterns (PAMPs), damage-associated molecular patterns (DAMPs), and neurotransmitters. The binding of these stimuli to their respective cell surface receptors, such as TNF receptor (TNFR), interleukin-1 receptor (IL-1R), Toll-like receptors (TLRs), and NOD-like receptors (NLRs), leads to receptor activation. Activation of the NF-κB pathway plays a crucial role in the development of various neurological disorders by triggering a signaling cascade. Upon receptor activation, signaling pathways converge on the IκB kinase (IKK) complex. The activated IKK complex subsequently phosphorylates the IκB proteins, leading to their ubiquitination and proteasomal degradation. This releases NF-kB dimers, typically composed of p50 and p65 subunits, which translocate from the cytoplasm to the nucleus, where NF-kB binds to specific DNA sequences known as κB sites, thereby activating the transcription of a wide array of proinflammatory genes involved in neuroinflammation. These genes encode cytokines (e.g., TNF-α, IL-1β, and IL-6), chemokines, adhesion molecules, enzymes, and other inflammatory mediators. The products of NF-kB target genes further amplify the inflammatory response by recruiting immune cells, promoting cytokine release, enhancing oxidative stress, and contributing to neuronal damage [133]. Certain miRNAs can directly target and inhibit the expression of NF-kB subunits or their associated proteins. For example, miR-146a acts as a critical negative regulator of TLR-4 signaling. When TLR-4 is activated by ligands such as lipopolysaccharide (LPS), it induces the expression of miR-146a. This forms a negative feedback loop that controls the duration and intensity of the inflammatory response mediated by TLR-4. miR-146 directly targets key signaling components of the TLR-4 pathway, including IRAK1 (Interleukin-1 receptor-associated kinase 1) and TRAF6 (TNF receptor-associated factor 6), leading to their downregulation.

The inhibition of NF-kB activation downstream of TLR-4 was achieved using this approach. Furthermore, miR-146a directly inhibits NF-kB activation by targeting its signaling components. By suppressing the expression of IRAK1 and TRAF6, miR-146a prevents the activation of the IKK complex and subsequent NF-kB activation. This creates a negative feedback loop that helps dampen the inflammatory response mediated by TLR-4 and NF-kB. The interplay between miR-146a, TLR-4, and NF-kB allows for the precise control of the inflammatory response. miR-146a serves as a key mediator in the maintenance of immune homeostasis by modulating the intensity and duration of TLR-4-induced NF-kB activation. The dysregulation of this chemistry can lead to excessive inflammation, contributing to the pathogenesis of various inflammatory diseases including neuroinflammation-related disorders. The dysregulation of miR-146a has been implicated in AD pathology [134,135]. MicroRNA-146a-5p, along with its associated genetic loci, exerts a crucial influence on the regulation of TMAO levels and may be implicated in the pathogenesis of cardiometabolic disorders, such as atherosclerosis [136]. The role of arteriosclerosis in neurodegenerative disease is well established [137,138]_._

Conversely, miR-155 plays a crucial role in regulating the expression of proinflammatory genes. In the context of neuroinflammation, miR-155 is upregulated in AD brains and is involved in the regulation of NF-kB signaling by targeting multiple components of this pathway. It promotes neuroinflammation and contributes to AD pathogenesis by modulating the expression of proinflammatory TNF-α and IL-6, thus activating NF-kB. Indeed, recent studies have shown that the upregulation of miR-155 in patients with AD is reduced by using substances with anti-inflammatory properties or anti-NF-κB agents, such as curcumin or caffeic acid phenethyl ester [139].

Other miRNAs have been validated as targets of the NF-kB pathway in the context of neurodegenerative diseases such as AD. For instance, miR-125b has been shown to target TRAF6 and inhibit NF-kB activation. The dysregulation of miR-125b has been observed in AD and its downregulation may contribute to increased NF-kB activity and neuroinflammation. Similarly, miR-9 regulates NF-kB signaling by targeting NF-kB1 and IKKβ, leading to the suppression of NF-kB activation. Altered miR-9 expression has been reported in AD, and its dysregulation may contribute to neuronal dysfunction. Moreover, miR-34a is upregulated in AD brains and targets SIRT1 (sirtuin 1) and Bcl-2 (B-cell lymphoma 2), leading to increased NF-kB activity and apoptosis. The dysregulation of miR-34a contributes to neuronal cell death and AD pathology. In addition, miR-21 is upregulated in AD and targets the phosphatase and tensin homolog and PDCD4 (Programmed cell death protein 4), leading to the activation of the NF-kB pathway and the promotion of neuroinflammation. Overall, these miRNAs represent a subset of the complex regulatory network involved in modulating NF-kB signaling in neurodegenerative diseases. The dysregulation of these miRNAs contributes to the neuroinflammation, synaptic dysfunction, and neuronal cell death observed in AD and other neurodegenerative conditions. Targeting these miRNAs has therapeutic potential for mitigating neuroinflammation and slowing the progression of neurodegeneration [140,141,142,143,144].

**Figure 2:**
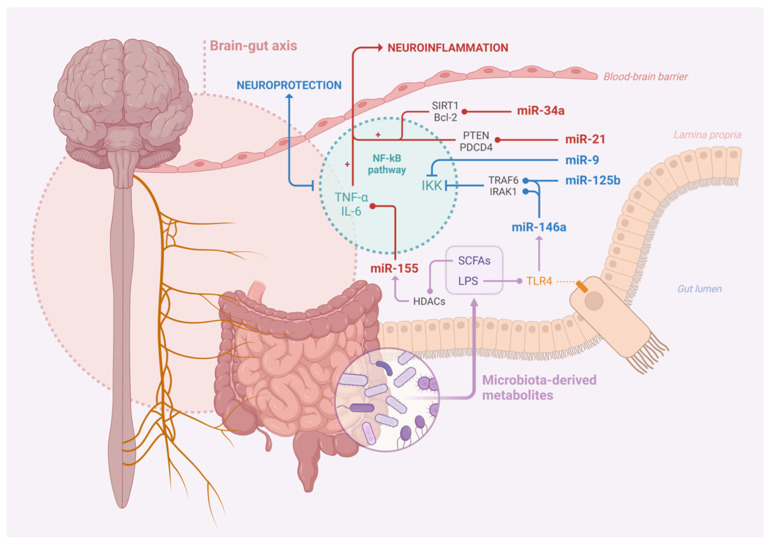
miRNA complex player between the gut and the brain.

Neurodegenerative diseases present significant challenges in terms of diagnosis and management because of their progressive nature and often overlapping clinical symptoms. As the search for reliable biomarkers intensifies, miRNAs have emerged as promising candidates because of their stability and potential to reflect the disease pathophysiology. In addition, miRNAs can be readily detected in bodily fluids such as blood, cerebrospinal fluid (CSF), and saliva. The use of miRNAs as biomarkers eliminates the need for invasive procedures, making them attractive candidates for routine diagnostic testing. As mentioned above, emerging evidence suggests that the gut microbiota influences miRNA expression in brain function and may contribute to the pathogenesis of neurodegenerative diseases. miRNAs have been implicated in mediating communication between the gut and the brain (Figure 2). Targeting these miRNAs as biomarkers allows the investigation of their roles in disorders of gut–brain interactions (DGBIs) and microbiota-mediated neuroinflammation [145,146].

### 4.3. Prevention and Treatment in Neurodegenerative Diseases

Modulating the gut microbiome has emerged as a potential therapeutic avenue for neurodegenerative diseases [147].

Various therapeutic approaches that target the gut microbiome include probiotics, antibiotics, dietary patterns, prebiotics, microbial metabolites, and fecal microbiome transplantation. These strategies can directly or indirectly modify the composition of the gut microbiome to potentially benefit patients with neurodegenerative diseases. It is important to note that these therapeutic approaches are not limited to the aforementioned list and that research is ongoing to identify other potential strategies for modulating the gut microbiome. Furthermore, the use of these approaches should be carefully considered and monitored by a healthcare professional to ensure their safety and efficacy. Individuals diagnosed with neurodegenerative diseases are likely to take medications to address accompanying pathologies that can influence the microbiome and the level of TMAO.

A well-balanced diet is crucial for the prevention and management of AD and PD.

The basic principles include reducing the consumption of red meat and consuming a diet rich in fruits, vegetables, whole grains, and legumes [148]. The production of short-chain fatty acids (SCFAs) occurs as a consequence of the bacterial fermentation of dietary fiber in the digestive system. These substances, which comprise acetate, propionate, and butyrate, are crucial for ensuring optimal gut health. This involves the regulation of mucus production, immune system function, and barrier function. In addition, SCFAs possess the ability to traverse the blood–brain barrier, where they can influence brain function through specific mechanisms [149,150].

SCFAs have therapeutic potential to alleviate AD and PD [151]. SCFAs, particularly sodium butyrate (SB), can protect neurons, improve memory functions, and induce cognitive improvement in AD model mice by increasing the expression of genes encoding neurotrophic factors [152].

Furthermore, SCFAs have demonstrated anti-neuroinflammatory effects, interfered with the formation of neurotoxic Aβ aggregates, and protected against Aβ-induced neurotoxicity [153].

In general, SCFAs have gained considerable interest as potential treatments for AD and PD owing to their capacity to influence neuroinflammation, synaptic plasticity, and Aβ accumulation. Further investigation is necessary to decipher the underlying mechanisms by which SCFAs impact AD and PD pathogenesis, as this could lead to the development of innovative therapeutic approaches that target gut microbiota-derived metabolites in the treatment of AD and PD.

Diet is important in reducing TMAO levels and improving the health state of the microbiome [154].

Cooking methods such as grilling may produce more TMAO than other methods, such as boiling or stewing. It is important to note that dietary interventions alone may not be sufficient to reduce TMAO and that statins can reduce the level of TMAO.

As matter of fact, Daniel Y Li et al. showed in a meaningful study that atorvastatin therapy (80 mgr. atorvastatin + 10 mgr. ezetimibe) reduces TMAO significantly [155]. Rosuvastatin reduces the ability of the intestinal flora to produce TMAO [156] and, in patients treated with statins, the therapeutic efficacy, expressed as the HDL/LDL ratio, correlates with TMAO plasma levels.

In recent years, nutraceuticals have assumed an important complementary role in the therapy of neurodegenerative diseases [157,158,159].

Resveratrol (3,5,4′ trihydroxystilbene, RSV) is a natural polyphenolic compound predominantly found in grapes, berries, and other dietary components. RSV has shown its utility in the treatment of various metabolic diseases, including arteriosclerosis, PD and AD [160,161,162].

RSV has been shown to reduce TMAO-induced proatherosclerosis by diminishing TMAO levels and increasing hepatic bile acid neosynthesis through the remodeling of the gut microbiota [163]. Additionally, RSV serves as a potent activator of sirtuins and counters the inhibitory action of TMAO on mitochondrial sirtuins and the activation of the cellular inflammasome [164]_._

Quercetin is a flavonoid that is commonly found in various fruits, vegetables, and nuts in a variety of glycosidic forms. Its principal food sources include lettuce, chili, blueberries, onions, capers, tomatoes, broccoli, and apples [165].

Quercetin possesses a range of beneficial effects such as antioxidant, anti-carcinogenic, anti-inflammatory, anti-microbial, anti-viral, anti-aging, anti-thrombotic, and vasodilatory properties [165].

Quercetin has been shown to reduce the levels of TMAO and its toxic effects in the liver [166].

Moreover, quercetin appears to improve and regulate the gut microbiota [167].

The enzyme beta-secretase is responsible for the production of beta-amyloid plaques at synaptic knobs. This enzyme plays a crucial role in the development of AD [168,169]. Molecular dynamics and simulation studies have revealed that Quercetin perfectly binds the binding pocket of beta-secretase enzyme hence brought inhibitory effect on its activity [170].

Quercetin is a promising agent in the prevention and treatment of neurodegenerative diseases [171]_._

Catechins, also known as flavan-3-ols, are a group of compounds found in plant-based sources. They possess potent antioxidant properties, which can help combat oxidative stress and inflammation. Additionally, these compounds have been shown to exhibit anti-diabetic, anti-cancer, anti-neuroprotective, bactericidal, and memory-enhancing effects. These various health benefits make catechins highly beneficial for overall health and well-being [172].

Catechins such as epigallocatechin (EGCG) possess iron-chelating properties that could be beneficial in neurodegenerative diseases like PD, where a misregulated iron metabolism plays a central role [173,174]. Moreover, research has demonstrated that catechins can promote the formation of non-toxic protein aggregates, which implies a positive influence on the aggregation pathways of neurodegenerative disorders such as Alzheimer’s disease and Parkinson’s disease [175].

Taurisolo, a dry extract obtained from the pomace of the Aglianico cultivar, contains standardized levels of resveratrol, catechins, and quercetin.

Studies have shown that Taurisolo has the ability to safeguard endothelial function from inflammatory processes and minimize ischemic brain injury in mouse models of ischemia and reperfusion [176].

In a double-blind, randomized, single-center, placebo-controlled, crossover study, Taurisol^®^ supplement was found to lower blood levels of TMAO [177]. In another randomized study, Taurisol^®^ lowered the TMAO level and improved the walking distance in patients with Rutheford class II. A possible explanation is the combined effect of polyphenols on remodeling the microbiota and the protective action on endothelial cells [178].

## 5. Conclusions

The relationship between the gut microbiome, gut–brain axis, and host metabolism in the development of neurodegenerative diseases such as Alzheimer’s disease and Parkinson’s disease is becoming increasingly apparent. Research suggests that the gut microbial metabolite trimethylamine N-oxide (TMAO) may play a significant role in the connection between gut dysbiosis and neurological dysfunction and neurodegeneration. Studies have found that elevated TMAO levels are associated with increased neuroinflammation, protein misfolding and aggregation, oxidative stress, and impaired neuronal signaling, which are all hallmark features of AD and PD. Furthermore, TMAO has the ability to cross the blood-brain barrier and directly impact brain pathology by promoting amyloid-beta and tau aggregation, disrupting the integrity of the blood-brain barrier, and inducing neuronal death.

It is intriguing to note that TMAO also modulates microRNA expression patterns, which are implicated in neurodegenerative processes. This gut microbiome-miRNA-brain axis represents a novel pathway through which gut dysbiosis may contribute to the initiation and progression of neurodegenerative conditions. Further elucidating these mechanisms could reveal potential therapeutic targets for miRNA-based interventions.

Integrating TMAO as a biomarker into diagnostic pathways alongside other molecular markers may improve the accuracy of early detection and disease monitoring for AD and PD. Harnessing the specific signatures of TMAO and associated miRNAs could facilitate the development of precision medicine approaches tailored to an individual’s gut microbiome composition and metabolic profile.

While significant challenges remain, modulating the gut microbiota through dietary interventions, pre/probiotics, or fecal transplants could offer novel strategies to restore gut–brain axis homeostasis and mitigate neurodegeneration. An integrative, multi-pronged approach targeting gut dysbiosis, TMAO levels, neuroinflammation, and microRNA dysregulation may unlock new therapeutic avenues for these debilitating conditions.

In summary, the pivotal role of the gut–brain axis and its microbial metabolites, particularly TMAO, in shaping the pathogenesis of neurodegenerative diseases has a solid background. Continued research efforts aimed at deciphering the intricate crosstalk between the gut microbiome, trimethylamine-N-oxide (TMAO), microRNAs, and brain function are essential for the development of effective preventive and therapeutic strategies against Alzheimer’s disease, Parkinson’s disease, and other related neurological disorders. It is crucial to continue these research efforts to develop effective treatments and preventative measures for these debilitating diseases.
